# Assessing the Prevalence of Depression Among English- and Spanish-Speaking Patients at a Free Medical Clinic

**DOI:** 10.7759/cureus.93648

**Published:** 2025-10-01

**Authors:** Anushka Parekh, Kushali Patel, Grace Ralston, Amanda Donald, Emma Beier, Lisa Carroll, David Redden

**Affiliations:** 1 Department of Family Medicine, Edward Via College of Osteopathic Medicine, Spartanburg, USA; 2 Department of Research and Biostats, Edward Via College of Osteopathic Medicine, Auburn, USA

**Keywords:** depression, healthcare disparities, language barrier, rural health disparities, spanish-speaking patients

## Abstract

Objective: The goal of this study was to assess the rate of positive depression screens in a randomly selected group of 111 patients at St. Luke’s Free Medical Clinic in Spartanburg, South Carolina, while also looking for differences in positive depression screeners between English- and Spanish-speaking patients. Our hypothesis is that at the free clinic, there is an overall low positive screening rate for depression in Spanish-speaking patients due to stigma, lack of interpreters, and the English language Patient-Health Questionnaire-2 (PHQ-2).

Methods: All patients seen at St. Luke’s fill out a PHQ-2, which screens for depression. Patients self-report this information. To conduct the research study, a random number generator was used to pick five dates from 2023 when patients were seen at the clinic. Patients were given a numerical code as deidentifiers, and the data were recorded in a password-protected computer. The deidentified data recorded included patient year of birth, biological sex, race, preferred language, need for an interpreter, depression screen date, depression screening result, and follow-up plan if positive for depression screening. Given the categorical nature of the outcomes, tests of association will be conducted with chi-square tests using a type I error rate of 0.05. All statistics and significance tests were performed using Statistical Analysis System 9.4 (SAS Inc., Cary, NC).

Results: Of the 111 patients, 20 individuals screened positive for depression (18.02%), while 90 screened negative (81.98%). Of the 20 individuals who screened positive, 10 (50.00%) had a documented follow-up plan. Using chi-square tests of association, those who screened positive for depression had a statistically significant association with English as their preferred language (13.03% positive given preferred language is English, 6.97% positive given preferred language is Spanish, p = 0.0175). Additionally, there was a statistically significant association between a positive depression screening and not needing an interpreter (13.761% positive given an interpreter was not needed, 6.2385% given an interpreter was needed, p = 0.0236).

Conclusion: Through our data collection and analyses, we observed a statistically significant difference in rates of positive depression screenings between English- and Spanish-speaking patients. This supports our hypothesis in theorizing that language barriers create inequities in access to healthcare and resources.

## Introduction

Language barriers in healthcare can lead to miscommunication between medical professionals and patients, resulting in a decreased quality of care [1]. Non-English speakers, therefore, face significant barriers in accessing medical care and resources [2]. A lack of interpreting services in primary care settings further potentiates this issue [3]. In these settings, the practice of informal interpreting, such as relying on the patient’s children or other family members to mediate communication, is very common; however, it is associated with a higher number of errors compared to professional interpreting [3]. Informal interpreting also raises ethical and confidentiality concerns, especially with sensitive topics such as mental health [3].

One of the rising mental health concerns today is depression, which is estimated to have a prevalence of 10%-15% worldwide [4]. Studies of depressive disorders have stressed the importance of the morbidity and mortality associated with depression [4]. Thus, early detection of depression is crucial to avoid future negative health outcomes. Underdetection of depression by primary care providers is approximately 50% for the general population, and this rate may be even higher for Latino immigrants, for whom the depression rate tends to be higher than in non-Hispanic Whites [5]. Research on the diagnostic accuracy of Spanish-language depression screening instruments remains scarce in the United States [5].

The Patient Health Questionnaire-9 (PHQ-9) has been widely validated for depression screening in primary care settings [6]. The PHQ-2 uses the first two questions of the PHQ-9 and is used as an initial screening tool. Its purpose is not to establish a final diagnosis or to monitor depression severity, but rather to screen for depression. Patients who screen positive should be further evaluated with the PHQ-9 to determine whether they meet criteria for a depressive disorder. The PHQ-2 has good sensitivity and specificity for detecting major depression, and due to the brief nature of the instrument, it is a good initial screening tool. Research on Spanish-speaking populations using a Spanish-validated PHQ-2 rather than an English-validated PHQ-2 continues to be scarce in the United States.

The purpose of this study is to analyze the rates of positive depression screenings among English- vs. Spanish-speaking patients in the primary care setting. We predict overall positive depression screening rates at the free medical clinic to be low, given the stigma surrounding mental health and the limited access to care. Additionally, looking at the Spanish-speaking patient populations, we expect this group to have an even lower screening positivity, due to the limited number of interpreters and the English format of the PHQ-2.

## Materials and methods

This study utilized a cross-sectional design. This study was approved as exempt by the Edward Via College of Osteopathic Medicine Institutional Review Board. All patients who are seen at the clinic fill out a basic information and demographic form that includes racial profile and language and interpretation preferences. All patients also fill out a PHQ-2 that screens for depression. The PHQ-2 is a two-item depression questionnaire, consisting of the first two questions from the PHQ-9. It was developed by Kroenke et al. [7]. To conduct the research study, a random number generator was used to pick five dates from 2023 when patients were seen at the clinic. These five dates were randomly selected to generate a feasible sample size of patients for detailed chart review. While this approach provides a representative subset of clinic patients, it may not fully capture seasonal variation or fluctuations in patient flow. These factors are acknowledged as limitations of the study design.

We gathered the names of the patients who were seen on these dates. If no patients were seen on one of these dates, a new date was randomly selected. The list of patient names was reviewed, and any duplicate names were deleted. Patients without a recent PHQ-2 (within two years) were excluded from the study sample. Patients were given a numerical code as deidentifiers, and a total of 111 patients were included in the final sample. The deidentified data recorded were the patient's year of birth, biological sex, race, preferred language, whether an interpreter was needed, the date of the depression screening, the depression screening result, and if a positive depression screening result was found, whether a documented follow-up plan was in place. The data were recorded in a password-protected computer.

A score of 3 is the optimal cutoff point when using the PHQ-2 to screen for depression [7]. This study uses the traditional score of 3 to indicate a positive depression screen (see Table 1 for question descriptions). The additional two questions about suicidality were implemented by St. Luke’s Free Medical Clinic to guide clinical judgment. While the inclusion of the two Yes/No questions deviates from the traditional PHQ-2 screen, which typically includes only the first two questions, these additional questions are useful to assess the severity of the situation and ensure timely intervention.

**Table 1:** PHQ-2 at St. Luke's Free Medical Clinic. This table demonstrates the PHQ-2 used for depression screening, including an additional two questions to assess suicidality. The criteria required to define a positive screening is a score of ≥3 Source: No permissions were required for use of this source. Pfizer states clearly that "All PHQ, GAD-7 screeners, and translations are downloadable from this website and no permission is required to reproduce, translate, display, or distribute them" PHQ-2: Patient Health Questionnaire-2

During the last two weeks, how often have you been bothered by any of the following problems	0	1	2	3
Little interest or pleasure in doing things	No days	3-5 days	7-10 days	Nearly every day
Feeling down, depressed, or hopeless	No days	3-5 days	7-10 days	Nearly every day
During the last two weeks, have you had thoughts of suicide or killing yourself?	Yes	No	-	-
Have you attempted suicide since your last visit to St. Luke's Free Medical Clinic?	Yes	No	-	-

Analyses began by summarizing the data with counts and proportions for categorical variables, such as demographic variables like race and sex, as well as outcomes of positive/negative screens for depression and the presence of a follow-up plan. For continuous measures, such as age, means and standard deviations were calculated. Two patients were excluded from the statistical analyses due to missing language preferences (n = 109). A third patient was excluded because they had missing information on both language preferences and racial profile (n = 108). Given the categorical nature of the outcome, tests of association were conducted with chi-square tests using a type I error rate of 0.05. All statistics and significance tests were calculated/performed using Statistical Analysis System 9.4 (SAS Inc., Cary, NC).

## Results

There were a total of 111 individuals in the sample. Of the 111, 41 individuals reported race as Caucasian, 28 reported race as African American, 39 reported race as Hispanic/Latino, and 3 reported race as other. Of the 111, 71 reported their preferred language as English, 38 reported their preferred language as Spanish, and 2 individuals had missing data and therefore were excluded from analyses. Of the 111, 34 required an interpreter, 75 did not require an interpreter, and two individuals had missing data and were therefore excluded from the analyses.

Of the 111, 20 individuals screened positive for depression (18.02%), while 90 screened negative (81.98%). Of the 20 individuals who screened positive, 10 have a documented follow-up plan. As shown in Tables 2, 3, screening positive for depression was associated with preferred language: 18 out of 71 (13.03%) English-speaking patients screened positive compared to two out of 38 (6.97%) Spanish-speaking patients. Screening positive for depression was also associated with the use of an interpreter: 18 out of 75 (13.761%) positive, given an interpreter was not needed, compared to 2 out of 34 (6.2385%) positive, given an interpreter was needed. Although not statistically significant at the 0.05 level, the proportion of positive screenings varied by self-reported race (5.1852% positive for African American, 7.5926% positive for Caucasian, and 7.2222% positive for Hispanic), resulting in a p value of 0.0874, as shown in Table 4.

**Table 2:** Test of association between depression and preferred language. The table compares depression-screening outcomes (positive vs. negative) across English- and Spanish-speaking language groups

Preferred language	Depression		
	No	Yes	Total
English	53 (57.97%)	18 (13.03%)	71
Spanish	36 (31.03%)	2 (6.97%)	38
Total	89	20	109
Overall p value	-	-	0.0175

**Table 3:** Test of association between depression and interpreter needed. The table demonstrates the relationship of depression screening results based on whether a language interpreter was required

Interpreter needed	Depression		
	No	Yes	Total
No	57 (61.239%)	18 (13.761%)	75
Yes	32 (27.761%)	2 (6.2385%)	34
Total	89	20	109
Overall p value	-	-	0.031

**Table 4:** Test of association between depression and self-reported race. The table demonstrates the depression screen positivity rates across Caucasian, African American, and Hispanic racial groups

Race	Depression		
	No	Yes	Total
African American	22 (22.815%)	6 (5.1852%)	28
Caucasian	30 (33.407%)	11 (7.5926%)	41
Hispanic/Latino	36 (31.778%)	3 (7.2222%)	39
Total	88	20	108
Overall p value	-	-	0.0874

## Discussion

The data demonstrate a statistically significant association between a positive depression screen and preferred language, with more English-speaking patients screening positive compared to Spanish-speaking patients. This difference may be attributed to the limited availability of trained medical interpreters at St. Luke’s Free Medical Clinic or the lack of a Spanish-language PHQ-2 available in the clinic. However, additional contributing factors such as cultural stigma surrounding mental health, barriers to accessing care, and varying levels of trust in healthcare providers may also play a role. While these hypotheses provide possible explanations, the authors acknowledge the limitations of the study in drawing definitive conclusions from this association.

In 2020, the Hispanic population in the United States reached 62.1 million, accounting for 19% of Americans, making it the nation’s second largest ethnic group behind White Americans and ahead of Black Americans [8]. English proficiency among the Latino population has risen substantially from 59% in 1980 to 72% in 2019 [8]. This means, however, that the remaining 28% of non-English speaking Latinos in the United States are at risk for decreased access to health care and quality of care due to language barriers [2]. Latino immigrants experience higher rates of depression and more depressive symptoms compared to non-Hispanic whites [9]. Despite this, Latino patients with Limited English Proficiency (LEP) have been found to have a lower likelihood of receiving mental health resources compared to their English-speaking counterparts, even when controlling for factors such as poverty, insurance status, and United States nativity [10].

Hypothesized reasons for these findings include the stigmatization of mental health in Latino cultures. Previous literature indicates that, within Hispanic populations, mental health is often stigmatized and viewed as a sign of personal weakness or interpreted through religious frameworks, such as divine punishment or a lack of faith [11]. Also observed is depression as a consequence of difficult life circumstances, failure in personal responsibilities, and immigrant-related pressures [12]. It is important for physicians to be aware of these connotations because they impact patients’ access to and willingness to seek care. Additionally, in immigrant populations, acculturation (the process of adapting to cultural elements of a dominant society) is associated with detrimental mental health symptoms [13]. This documented mistrust has led to poorer health outcomes and low healthcare utilization by Hispanic patients [14]. This is further corroborated by evidence that Hispanic patients are more likely to seek mental health care at primary healthcare clinics and emergency departments rather than at specialized mental health facilities [2,15]. Because individuals are more likely to seek services at primary care facilities, guidelines and funding in rural areas warrant efforts to support mental health services in primary care clinics [16].

To increase the quality of care for Spanish-speaking patients, reliable access to linguistic interpreters, validated screening tools, and provider awareness are necessary. Barriers to providing care for LEP Spanish speakers in primary care settings include having a small local population of Latinos, financial strain, and a lack of bilingual workers [17]. The most common forms of interpreting services include telephonic interpreting and using bilingual family members [17]. Interpreting services are common and user-friendly; however, in primary care settings, they are limited in relation to the large need for them [3]. While relying on family members for interpretation may be necessary in certain situations, untrained interpreters can misinterpret or omit important critical information [17]. Therefore, medical providers should minimize this practice in order to improve the quality of care for patients with LEP [17].

Future actions should be taken to ensure reliable access to telephone or video interpreting, provide education in cultural differences, and foster an environment where patients are more inclined to seek mental health treatment. This effort is especially needed in free clinics, where most of the patient population is underserved. While several depression screening tools have been translated into Spanish, there is limited research on their validity in the Spanish-speaking Latino population [5,18]. We hypothesize that implementing Spanish-language PHQ-2 screening forms may encourage more honest responses from Spanish-speaking patients if they are able to complete the form confidentially [19]. Especially for cultural groups that value privacy, self-reporting would encourage patients to honestly report their symptoms without embarrassment or feelings of being judged [20]. This could also apply to English-speaking patients, as individuals may generally be more forthcoming with personal information, such as feelings of depression, when answering these questions privately. A surprising finding during data collection was that out of the 20 positive depression screens, only 10 had a documented follow-up plan, leaving the other 10 without one. Chart reviews of these 10 cases revealed that the positive screening results were not addressed in the visit summaries. Unfortunately, it is unclear whether providers discussed the results with patients verbally but failed to document the conversation and any follow-up plans. Ideally, all patients with a positive depression screen should have a clearly documented follow-up plan to ensure continuity of care, particularly regarding mental health evaluation and treatment.

This study has limitations due to its small sample size, which makes it difficult to extrapolate the data to other geographical areas. The cross-sectional design precludes any conclusions regarding causality. In addition, studying patients from a free clinic limits the generalizability of the data to all income levels and socioeconomic statuses. Future research can be conducted with a larger population of Spanish speakers and may also include other commonly spoken languages, such as Chinese and Russian. Given the widespread use of the PHQ-2, further research is needed among Spanish-speaking patients to validate the clinical accuracy of the Spanish-language PHQ-2. Targeted interventions may include implementing a Spanish-language PHQ-2 and assessing the accuracy of depression screening results among Spanish-speaking patients when using the English versus the Spanish version.

## Conclusions

In conclusion, these data support a significant difference in rates of positive depression screenings based on spoken language. This finding could represent possible inequities in healthcare given to Spanish-speaking patients. Following the results of this study, recommendations were made to include a Spanish PHQ-2 at St. Luke’s Free Medical Clinic. After this implementation, a future study could be conducted to assess if depression rates change. Further research is needed to increase access to care for Spanish-speaking patients and mitigate the language barrier's impact on quality of care.
